# Multidimensional individualized nutritional therapy for individuals with severe chronic obstructive pulmonary disease: study protocol for a registry-based randomized controlled trial

**DOI:** 10.1186/s13063-023-07099-1

**Published:** 2023-02-06

**Authors:** Maria H. Hegelund, Christian Ritz, Thyge L. Nielsen, Mette F. Olsen, Christian Søborg, Lone Braagaard, Christian Mølgaard, Rikke Krogh-Madsen, Birgitte Lindegaard, Daniel Faurholt-Jepsen

**Affiliations:** 1 Department of Pulmonary and Infectious Diseases, Copenhagen University Hospital, North Zealand, Dyrehavevej 29, 3400 Hillerød, Denmark; 2 National Institute of Public Health, Copenhagen, Denmark; 3 Department of Infectious Diseases, Copenhagen University Hospital, Copenhagen, Rigshospitalet Denmark; 4 Department of Nutrition, Exercise and Sports, University of Copenhagen, Frederiksberg, Denmark; 5 Department of Infectious Diseases, Copenhagen University Hospital, Hvidovre, Denmark; 6 Center for Physical Activity Research, Rigshospitalet, Copenhagen University Hospital, Copenhagen, Denmark

**Keywords:** Chronic obstructive pulmonary disease, Quality of life, Nutritional status, Nutritional therapy, Mental health, Body composition, Functional capacity

## Abstract

**Background:**

Individuals with severe chronic obstructive pulmonary disease (COPD) are often at risk of undernutrition with low health-related quality of life (HRQoL). Undernutrition can worsen COPD and other comorbidities, be an independent predictor of morbidity and functional decline resulting in increased healthcare consumption and increased risk of death. Especially exacerbations and acute infections result in unintentional weight loss. The aim is to investigate the effect of an individualized nutritional intervention among individuals with severe COPD.

**Methods:**

An open-label randomized controlled trial with two parallel groups. Participants are recruited from the pulmonary outpatient clinic at the Department of Pulmonary and Infectious Diseases, Copenhagen University Hospital, North Zealand, Denmark, and randomly allocated to either the intervention (intervention + standard of care) or control group (standard of care). The intervention has a duration of 3 months and combines individual nutritional care with adherence support and practical tools. It contains 4 elements including an individual nutritional plan, regular contacts, adherence support, and weight diary. The primary outcome is a difference in HRQoL (EQ-5D-5L) between the intervention and control group 3 months after baseline. Difference in functional capacity (grip strength, 30-s stand chair test, and physical activity), disease-specific quality of life (COPD Assessment Test), anxiety and depression (Hospital Anxiety and Depression Scale), nutritional parameters (energy and protein intake), anthropometry (weight, body mass index, waist, hip, and upper arm circumference), body composition (total fat-free and fat mass and indices), and prognosis (exacerbations, oxygen therapy, hospital contacts, and mortality) 3 months after baseline will be included as secondary outcomes. Data will be collected through home visits at baseline and 1 and 3 months after baseline.

**Discussion:**

Currently, nutritional care is a neglected area of outpatient care among individuals with severe COPD. If this patient-centered approach can demonstrate a positive impact on HRQoL, mortality, and hospital contacts, it should be recommended as part of end-of-life care for individuals with severe COPD.

**Trial registration:**

ClinicalTrials.gov NCT04873856 . Registered on May 3, 2021.

## Administrative information

**Table:** 

Title {1}	Multidimensional individualized nutritional therapy for individuals with severe chronic obstructive pulmonary disease – study protocol for a randomized controlled trial MINDNUT
Trial registration {2a and 2b}	ClinicalTrials, NCT04873856
Protocol version {3}	Version 3 December 13, 2021
Funding {4}	Grosserer L. F. Foghts Fond Axel Muusfeldts Fond The Research Council at Copenhagen University Hospital, North Zealand, Denmark
Author details {5a}	**Maria H. Hegelund,** Department of Pulmonary and Infectious Diseases, Copenhagen University Hospital, North Zealand, Denmark **Christian Ritz**, National Institute of Public Health, Copenhagen, Denmark **Thyge L. Nielsen**, Department of Pulmonary and Infectious Diseases, Copenhagen University Hospital, North Zealand, Denmark **Christian Mølgaard**, Department of Nutrition, Exercise and Sports, University of Copenhagen, Frederiksberg, Denmark **Mette Frahm Olsen**, Department of Infectious Diseases, Copenhagen University Hospital, Rigshospitalet, Copenhagen, Denmark Department of Nutrition, Exercise and Sports, University of Copenhagen, Frederiksberg, Denmark **Christian Søborg**, Department of Pulmonary and Infectious Diseases, Copenhagen University Hospital, North Zealand, Denmark **Lone Braagaard**, Department of Pulmonary and Infectious Diseases, Copenhagen University Hospital, North Zealand, Denmark **Rikke Krogh-Madsen**, Department of infectious Diseases, Copenhagen University Hospital, Hvidovre, Denmark Center for Physical Activity Research, Copenhagen University Hospital, Rigshospitalet, Copenhagen, Denmark **Birgitte Lindegaard**, Department of Pulmonary and Infectious Diseases, Copenhagen University Hospital, North Zealand, Denmark Center for Physical Activity Research, Copenhagen University Hospital, Rigshospitalet, Copenhagen, Denmark **Daniel Faurholt-Jepsen**, Department of Infectious Diseases, Copenhagen University Hospital, Rigshospitalet, Copenhagen, Denmark
Name and contact information for the trial sponsor {5b}	***Daniel Faurholt-Jepsen****,* daniel.faurholt-jepsen@regionh.dk + 45 26 74 12 42
Role of sponsor {5c}	The sponsor is involved in study design, data collection, data management, data analysis, interpretation of the results and in deciding which journals manuscripts will be submitted to. The funder will have no role in study design, data collection, data management, data analysis, interpretation of the results and in deciding which journals manuscripts will be submitted to

## Introduction

### Background and rationale {6a}

Chronic obstructive pulmonary disease (COPD) is a frequent chronic disease and a serious global health concern that imposes significant burdens for individuals and societies. In Denmark, an estimated number of 50,000 individuals are living with severe COPD [1], and COPD is the third leading cause of death with the highest mortality rate in the European Union [2]. Each year, 25,000 hospitalizations have COPD as the primary diagnosis, and hospitalization due to COPD is related to a high risk of mortality with 7% dying within a hospital and 25% within the first year after discharge [1]. Severe COPD is associated with a high symptom burden, increased risk of anxiety, and depression, all with a negative impact on health-related quality of life (HRQoL) [3].

COPD is also closely linked to undernutrition, a state of malnutrition in which the intake of energy and protein falls below the biological needs, and in individuals with severe COPD, undernutrition is likely caused by several factors including metabolic and pathophysiological changes as well as social and mental conditions [4]. Undernutrition can worsen COPD and lead to increased morbidity and functional decline resulting in increased healthcare consumption and risk of death [4]. Inadequate dietary intake is common among individuals with COPD [5]. Especially pulmonary exacerbations and acute infections result in unintentional weight loss [6]. Undernutrition is associated with decreased HRQoL [7], low physical capacity [8], and mortality [9].

Comorbidities such as anxiety and depression are common but have frequently been overlooked because the symptoms overlap the symptoms related to COPD itself. Without recognition and medical treatment, depression and anxiety symptoms may have serious negative effects on HRQoL, functional capacity, and social interaction as well as increasing fatigue, more frequent exacerbations, and hospitalizations [10, 11].

Systematic reviews have suggested that nutritional support in COPD improves nutritional intake, anthropometric measures, functional capacity, and HRQoL. Many of the studies included in these reviews are conducted more than 30 years ago and based on small sample sizes. In addition, the interventions were not individualized, only a few studies focus on outpatients exclusively with severe COPD, and prognostic outcomes such as hospitalizations and mortality are very sparse [12, 13]. Nutritional care is an important but neglected area in the care of COPD likely due to knowledge gaps and the absence of professional responsibility [14]. There is a need for developing strategies to handle this underprioritized and complex problem in severe COPD. This paper describes a study protocol for a registry-based randomized controlled superiority trial investigating the effect of an intervention combining nutritional care with socio-emotional and adherence support and practical tools for improving HRQoL as well as nutritional status and prognosis in individuals with severe COPD.

### Objectives {7}

The objective is to investigate the effectiveness of multidimensional individualized nutritional therapy with a duration of 3 months on HRQoL as well as several clinical outcomes.

Primary outcome:

1. HRQoL

Secondary outcomes:

2) Hospital contacts
3) Mortality
4) Disease-specific quality of life
5) Anxiety and depression
6) Functional capacity
7) Anthropometry
8) Body composition
9) Exacerbations
10) Physical activity
11) Energy and protein intake
12) Difference between protein and energy requirement and intake

### Trial design {8}

The study is a single-center, home-based, effectiveness, registry-based randomized controlled superiority trial with two parallel groups.

## Methods: participants, interventions, and outcomes

### Study setting {9}

The recruitment takes place at the Department of Pulmonary and Infectious Diseases, Copenhagen University Hospital, North Zealand, Denmark. All data collection is carried out through home visits, telephone calls or medical records.

### Eligibility criteria {10}

When potential participants are identified, the study coordinator will screen them for eligibility.

Inclusion criteria

-  ≥ 35 years
- Severe COPD defined as GOLD grade 2 group B or D OR GOLD grade 3 and 4 group ABCD [15]
- Able to eat orally
- Live in own home
- Speak Danish or English
- Undernourished (BMI < 18.5 kg/m.^2^) OR at risk of undernutrition (a maximum BMI of 25 kg/m2) determined by Nutritional Risk Screening 2002 (NRS2002) or Mini Nutritional Assessment short form (MNA-SF)
- Stable phase

Exclusion criteria

- “Active solid cancer” defined as cancer diagnosed within the previous 6 months, recurrent, regionally advanced, or metastatic cancer, cancer for which treatment had been administered within 6 months or hematological cancer that is not in complete remission [16].
- Unable to sign informed consent, e.g., due to severe dementia.
- Severe chronic renal failure defined as estimated glomerular filtration rate < 30 mL/min./1.73 m^2^ (ICD-10 codes N18.4 and N18.5).
- Severe alcohol abuse (ICD-10 codes F10.2 and K70.x).

Drop out criteria.

- Lost to follow-up
- Withdrawal

### Daily management

All contact with the participants will be coordinated and conducted by the study coordinator.

### Who will take informed consent? {26a}

The written informed consent will be obtained at the first home visit prior to baseline data collection by the study coordinator.

### Additional consent provisions for collection and use of participant data and biological specimens {26b}

Participants are informed that signing the informed consent gives the researchers permission to save and store their personal information and data for future research on the effect of multidimensional individualized nutritional therapy. This study does not involve collection of biological material.

## Interventions

### Explanation for the choice of comparators {6b}

Standard of care augmented with a multidimensional individualized nutritional therapy will be compared to the standard of care. It is the intention that this study will highlight the importance of enhancing the focus of HRQoL, nutritional, socio-emotional, and adherence support compared to the current standard of care.

### Intervention description {11a}

#### Public and patient involvement

There was no public or patient involvement in the design of this protocol.

#### Nutritionally augmented standard of care

The intervention has been designed by a multidisciplinary team of experts within pulmonary medicine, infections, nutrition, and physical (in)activity. The intervention has a duration of 12 weeks. In addition to standard of care, the intervention contains four elements including (1) individual nutritional plan, (2) regular contact, (3) adherence support, and (4) weight diary. The four elements are described below:

- *Nutritional plan* is developed and adjusted with the participant based on nutritional registration, information about routines and habits and nutritional preferences to reach a daily protein target of 1.5 g protein/kg/day [17] and individual energy requirements of 30–45 kcal/kg/day [17]. The nutritional plan is adjusted at the regular contacts. The nutritional plan may include supplementation with energy and protein-rich products and during the intervention period, a document summarizing tools and advices are provided.
- *Regular contacts* are phone calls from the project coordinator every 7–14th day to talk about the nutritional plan, well-being, adherence, or potential side effects.
- *Adherence support* is provided through the regular contact and a friendly reminder that encourages the participant to follow the nutritional plan and to ask for support (a note to hang on the refrigerator).
- *Weight diary* is a daily registration of weight.

Note: It is likely that participants in both groups will be hospitalized during the intervention period. As far as possible the study coordinator will continue the intervention during hospitalization. Hospitalizations with acute infections such as pneumonia and exacerbation are common among individuals with severe COPD and both conditions increase nutritional requirements. It is therefore essential to maintain the contact with nutritional and mental support during hospitalization.

### Standard of care

At the Department of Pulmonary and Infectious Diseases at Copenhagen University Hospital, North Zealand, each patient is assigned with a nurse and offered an annual conversation about advance care planning. The purpose of advance care planning is to talk about disease severity, prognosis, treatment options, symptom burden, and preparation for the late stages and death. The nurses have the responsibility to maintain a personal relationship and are available by phone every working day with the possibility to get an appointment within 2 working days. A rehabilitation program combining physical training and education on all aspects of COPD is offered every second to third year, though the rehabilitation program has a very limited focus on nutrition.

### Criteria for discontinuing or modifying allocated interventions {11b}

The risk of side effects related to intake of nutritional supplementation is considered minimal and potential side effects is expected to be mild, primarily in the form of gastrointestinal symptoms. There are no known long-term risks.

### Strategies to improve adherence to interventions {11c}

The strategies to improve adherence to the intervention are integrated in the design in different ways. First, the adherence support and the regular contacts encourage the participant to follow the nutritional plan and remind the participant of the opportunity to ask for help and support. The regular contact also allows for adjusting the plan to improve adherence if the participant experience challenges in reaching the nutritional targets as the nutritional intake will be registered regularly during the intervention period. In addition, this conversation is also an opportunity for the participant to talk about well-being, potential side-effect or other challenges or issues or topics of interest.

### Relevant concomitant care permitted or prohibited during the trial {11d}

The participants can receive all forms of care. We encourage to act and live as normal as possible during this trial. Contact with the outpatient clinic and the hospital will be registered.

### Provisions for post-trial care {30}

The participants will not receive any post trial care beyond standard of care.

### Outcomes {12}

#### Primary outcome

The primary outcome is HRQoL measured using the EuroQoL EQ-5D-5L questionnaire [18], a generic health status questionnaire that has been validated in individuals with COPD [19].

#### Secondary outcomes

Secondary outcomes are weight and BMI, total fat-free mass, fat mass, and indices measured 3 months after baseline. Disease-specific quality of life measured as mean total score 1 and 3 months after baseline. Functional capacity measured as grip strength [20] and the 30-s chair stand test measured 1 and 3 months after baseline [21]. Hospital contact (outpatient contact by phone or visits, unplanned acute visit to the emergency ward (< 6 h), and unplanned acute hospital admission) is measured as the number of contacts between baseline 1 and 3 months after baseline. In addition, hospital admissions will also be measured as total days spent in the hospital during the 3-month study period. Mortality will be measured as percentage of individuals who died between baseline and end of the study period (3 months after baseline).

#### Other outcomes

Other outcomes are waist, hip, and upper arm circumference. Exacerbations are measured as the number of exacerbations between baseline and 3 months after baseline. Oxygen therapy is measured as percentage using home oxygen 3 months after baseline. Anxiety and depression as percentage of individuals with symptoms of any of the two conditions. Physical activity level is assessed with a questionnaire and with accelerometers 7 days from baseline and 7 days from the 3-month follow-up visit. Total protein and energy intake will be used as nutritional outcomes. In addition, for energy and protein, the difference between intake and requirement will be another outcome. Requirements are defined as follows: The protein target for all participants is defined as 1.5 g protein/kg/day [17], whereas energy requirements will be individually evaluated according to age and BMI. The energy requirements will be defined as 30 kcal/kg/day for weight maintenance and 45 kcal/kg/day for weight gain [17]. For participants < 70 years with BMI < 20 kg/m^2^ and participants ≥ 70 years with BMI < 22 kg/m^2^ the goal is weight gain [22], whereas the goal is weight maintenance for the remaining participants.

### Participant timeline {13}

**Fig. 1:**
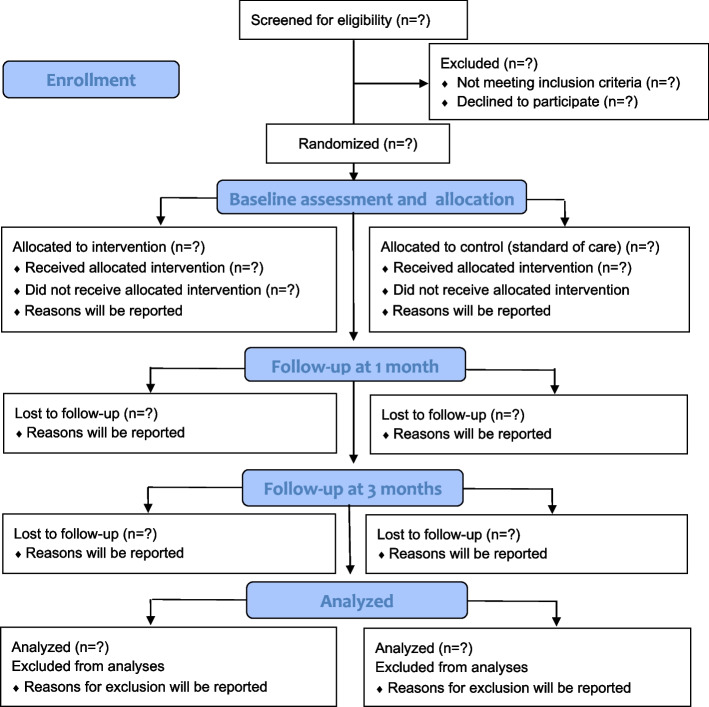
Flow of participants

The participant timeline is shown in Table 1 and the flow diagram in Fig. 1. All participants will have a home visit to collect data at baseline and 1 and 3 months after baseline. Enrollment began in May 2021 and is estimated to end in March 2023.

**Table 1:**
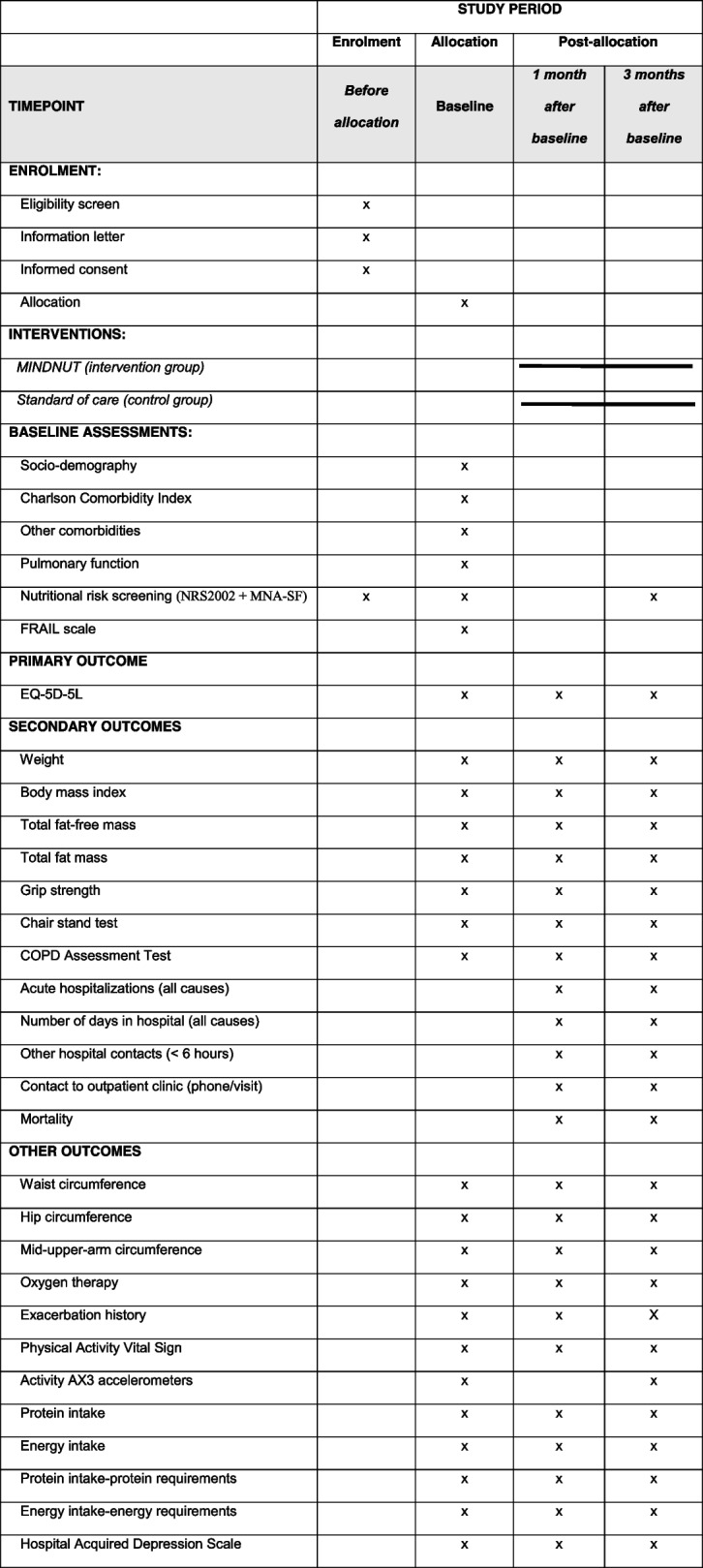
SPIRIT standard protocol items with participant schedule of enrolment, interventions, and assessments

The table is adapted based on the SPIRIT recommendations [23]

### Sample size {14}

In our study, the primary outcome, HRQoL, is measured using the EuroQoL EQ-5D-5L questionnaire. However, our sample size is calculated based on results from a Japanese study investigating the effect of nutritional support combined with exercise on several outcomes including HRQoL measured using the chronic respiratory disease questionnaire among stable COPD patients. The chronic respiratory disease questionnaire consists of four domains (dyspnea, fatigue, emotional, and mastery) with 20 items in total. Each item is scored on a 7-point scale. There are no reports of a clinical relevant change/difference in the total HRQoL score of the chronic respiratory disease questionnaire. In the Japanese study, a 10-point higher total HRQoL score was reported in the intervention group compared to the control group (109 vs. 99, *p* = 0.04) at the end of the intervention with a standard deviation of 17 points [24] as used in our sample size calculation. With an estimated drop-out rate of 20%, the inclusion of 60 participants in each group, will provide a power of 0.8 at a significance level of 0.05.

### Recruitment {15}

Participants are recruited from the pulmonary ward and the outpatient clinic at the Department of Pulmonary and Infectious Diseases, Copenhagen University Hospital, North Zealand, Denmark. Potential participants are identified in the following three ways: (1) lists of individuals with COPD followed in the pulmonary outpatient clinic, (2) hospitalized in the pulmonary ward, or (3) through visits in the pulmonary outpatient clinic. The list of individuals with COPD is generated by a person from the analysis team at the hospital from the electronic medical journal (Sundhedsplatformen, EPIC, USA) and contains individuals in contact with the pulmonary outpatient clinic within a defined period (e.g., the last 12 months) with IC-10 diagnosis codes from DJ439 to DJ449. Potential participants are chosen from the generated list using random numbers generated by the statistical program R [25]. The randomly chosen individual is screened for eligibility and participants with potential eligibility will receive a letter with information about the project. Recruitment from the list will be the primary recruitment method. If it is considered necessary to use supplemental recruitment methods, the other two methods will be used. The study coordinator will visit patients during a hospitalization in the pulmonary ward and shortly introduce the project through a short oral presentation and the information letter. Finally, the nurses from the outpatient clinic have been encouraged to hand over the information letter during visits when an individual is considered potentially eligible (BMI < 25 kg/m^2^) to participate in the project. All identified potential participants will receive a phone call from the study coordinator with further information about the project.

After receiving an oral informed consent and if the inclusion criteria are met, a home visit will be scheduled. At the first home visit, informed written consent will be obtained, baseline data will be collected, and group allocation will be revealed in the abovementioned order.

## Assignment of interventions: allocation

### Sequence generation {16a}

Participants will be randomly allocated to either intervention or control group using computer-generated block randomization with random group sizes of 2, 4, and 6 (using the R extension package “blockrand”) [26].

### Concealment mechanism {16b}

A file/photo of each allocation by ID number will be saved in a separate folder on a secure network drive. When a participant is enrolled in the study, the study coordinator will open the ID number and the allocation will be revealed.

### Implementation {16c}

The allocation sequence is performed by a person not involved in the project. This person has also performed the concealed allocation by saving a file/photo of each allocation by ID number at a secure folder at a network drive at the hospital. The study coordinator who is blind for the allocation will enroll participants. After the baseline assessment, the group allocation will be revealed for the study coordinator and the participant.

## Assignment of interventions: blinding

### Who will be blinded {17a}

This is an open-label study. The trial coordinator is responsible for all parts of the project including intervention, data collection, data analysis, and dissemination. Though the trial coordinator is blinded during the baseline assessment and data analyses.

### Procedure for unblinding if needed {17b}

Since blinding is not possible after baseline assessment unblinding will not be needed.

## Data collection and management

### Plans for assessment and collection of outcomes {18a}

An overview of the assessment and collection of outcome variables is shown in Table 2. All data collection will be performed by the study coordinator or under special circumstances other trained personnel (a project nurse or a student assistant).

**Table 2:** Overview of study outcomes

Assessments	Specific measurements	*Timepoint*			*Mean/median*	*Percent/proportion*	*Type of variable*
		***Baseline***	***1 mo***	***3 mos***			
**Primary outcome**							
Health-related quality of life	EQ-5D-5L	x	x	x	x		Numerical
**Secondary outcomes**							
Hospitalizations	Acute hospitalizations (all causes)		x	x		x	Numerical
Days in hospital	Number of days in hospital (all causes)		x	x	x		Numerical
Other hospital contacts	Acute hospital contacts < 6 h		x	x	x		Numerical
Contact to the outpatient clinic	Contact to the outpatient clinic via phone or visit		x	x	x		Numerical
Mortality	Mortality (number of deaths)		x	x		x	Binomial
Disease-specific quality of life	COPD Assessment Test	x	x	x	x		Numerical
Anxiety and depression	Hospital Acquired Depression Scale	x	x	x		x	Binomial
Muscle strength	Grip strength	x	x	x	x		Numerical
Muscle endurance	30-s chair stand test	x	x	x	x		Numerical
Fat-free mass	Bioelectrical impedance analysis	x	x	x	x		Numerical
Fat mass	Bioelectrical impedance analysis	x	x	x	x		Numerical
Weight	Electronic scale	x	x	x	x		Numerical
Body mass index	Weight (kg)/height(m^2^)	x	x	x	x	x	Numerical/categorical
**Other outcomes**							
Hip circumference	Measuring tape	x	x	x	x		Numerical
Waist circumference	Measuring tape	x	x	x	x		Numerical
Mid-upper arm circumference	Measuring tape	x	x	x	x		Numerical
Exacerbation history	Number of exacerbations	x	x	x	x		Numerical
Oxygen therapy	Yes/no	x	x	x		x	Binomial
General physical activity level	Physical Activity Vital Sign	x	x	x	x		Numerical
Physical activity level	AX3 accelerometers	x		x	x		Numerical
Protein intake	24-h recall	x	x	x			Numerical
Energy intake	24-h recall	x	x	x			Numerical
Difference between energy requirement and intake	24-h recall	x	x	x			Numerical
Difference between protein requirement and intake	24-h recall	x	x	x			Numerical

#### Baseline assessment and study questionnaires

At the baseline visit information about socio-demography, pulmonary function, oxygen use, exacerbation history (prior 12 months), comorbidities including Charlson Comorbidity Index modified by Quan [27], drinking and smoking habits are collected through questionnaires and medical records. At the baseline visit, the following questionnaires will be answered in collaboration with the participant: NRS2002 [28] and MNA-SF [29] (nutritional risk), EuroQoL EQ-5D-5L (HRQoL) [18], Hospital Acquired Depression Scale (anxiety and depression) [30], COPD Assessment Test (disease-specific quality of life) [31], FRAIL scale (frailty) [32], The Physical Activity Vital Sign (physical activity) [33], 24-h recall (dietary registration) [34]. At the 1-month follow-up visit the following questionnaires will be repeated: EuroQoL EQ-5D-5L, Hospital Acquired Depression Scale, COPD Assessment Test, The Physical Activity Vital Sign, 24-h recall. At the 3-month follow-up, the following questionnaires will be repeated: Nutritional Risk Screening 2002, EuroQoL EQ-5D-5L, Hospital Acquired Depression Scale, COPD Assessment Test, The Physical Activity Vital Sign, and 24-h recall (dietary registration).

#### Nutritional risk screening

Nutritional risk is determined using NRS2002 and MNA-SF as part of the recruitment process, at the baseline and 3-months follow-up visits. The NRS2002 is largely used in the hospital setting, whereas MNA is recommended among elderly and frail individuals. The purpose is to provide simple screening tools to detect individuals at risk of undernutrition who might benefit from nutritional support. In the NRS2002, the nutritional risk is defined by the present nutritional status and risk of impairment of present status, due to increased requirements caused by stress metabolism of the clinical condition. The NRS2002 score determines if the patient is at nutritional risk using a scoring system including information about BMI, weight loss, reduced food intake, disease severity, and age. The patient is at nutritional risk if the total score is ≥ 3 [28]. In addition, the MNA-SF is used as supplemented information on nutritional risk. MNA also determines nutritional status based on a scoring system including information on food intake, weight loss, mobility, psychological stress, neuropsychological problems, and BMI. A total score of 12–14 indicates normal nutritional status, whereas a total score of 8–11 and 0–7 indicate risk of malnutrition and malnutrition, respectively [28].

#### Diagnosis of COPD, pulmonary function, and disease severity

The diagnosis of COPD is based on spirometry with a post-bronchodilator FEV_1_/FVC < 0.70 plus symptom burden and risk of exacerbation (group A to D) according to the refined GOLD ABCD assessment tool [15]. Severe COPD is defined as all patients with GOLD grades 3 and 4 (airflow limitation FEV_1_ < 50% of the predicted value) and individuals with GOLD grade 2 with a high symptom and/or exacerbation burden (group B or D). A high symptom burden is based on a CAT score ≥ 10 or a score ≥ 2 on the modified MRC Dyspnea Scale [15]. Information about airflow limitation (pulmonary function) will be obtained from patient files. At baseline and 12 months after baseline, the latest pulmonary function assessment will be registered.

#### COPD assessment test

The COPD Assessment Test contains 8 items with a scoring range of 0–40. The total score indicates the impact level COPD has on the everyday life ranging from low to very high. A total score of 5 is referred to the upper limit of normal in healthy non-smokers, a total score < 10 indicates low impact, 10–20 indicates medium impact, whereas > 20 and > 30 indicate high and very high impact, respectively [31, 35].

#### Health-related quality of life

HRQoL is assessed using the EuroQoL EQ-5D-5L questionnaire [18, 19]. The questionnaire consists of five categories/questions including mobility, self-care, daily activities, pain/discomfort, and anxiety/depression. For each question, there are five possible answers each containing a score from 1 (no problems) to 5 (extreme problems). This will provide a combination of numbers [18] which is translated to a country-specific total index [36]. The lowest Danish index score is − 0.624 and the highest score is 1.000. In addition, the participant is asked to evaluate their own health on a scale from 0 to 100. A score of 100 means the best imaginable health [18].

#### Depression and anxiety

The risk of depression and anxiety will be determined using Hospital Acquired Depression Scale (HADS). HADS contains two subscales to evaluate the risk of anxiety and depression. Each subscale contains 7 items with a total score from 0–21. Risks of anxiety and depression are based on a total score ≥ 8 and a total depression score ≥ 8 or ≥ 5 (if the scale is reduced with one item), respectively [30].

#### Anthropometry and body composition

Height (cm) is based on the latest measurement in their medical record and confirmed by the participant. Weight is measured to the nearest 0.1 kg on an electric scale (TANITA DC 430 SMA, TANITA, Denmark). Body mass index (BMI) is calculated as weight (kg)/height (m^2^). Waist, hip, and mid-upper arm circumference will be measured to the nearest 0.1 cm. Waist circumference will be measured at the midline between the lowest border of the rib cage and the upper border of the iliac crest. Hip circumference will be measured at the broadest level on the hips while participants are standing [37]. Upper-arm circumference will be measured at the right arm at the mid-point between the acromion and the olecranon [38] in sitting or standing posture. Bio-impedance (TANITA DC 430 SMA, TANITA, Denmark) will be used to assess the distribution of total fat-free, fat mass, and indices.

#### Nutritional intake

Nutritional intake will be assessed using 24-h recall [34]. Total protein and energy intake will be calculated using MADlog (MADLOG, Denmark), which is a tool that calculates energy and protein from registered food intake. Protein intake is measured as grams and calories of protein and energy consumed within 24 h, respectively.

#### Functional capacity

##### Muscle strength

Grip strength will be used as an indicator of overall muscle strength and will be measured using a hand dynamometer (SAEHAN DHD-1 Digital Hand Dynanometer, SAEHAN Corporation, South Korea). During the test, the participant will be sitting or standing with the upper arm held along the body with the elbow bent 90 degrees, while the wrist is kept in a neutral position [39]. The test is performed repeated three times on the dominating hand and the highest value is registered. If the participant is uncertain about which hand is strongest, the test will be performed three times on each hand and the highest of all measurements is registered Grip strength is measured in kg.

##### 30-s chair stand test

The 30-s chair stand test is used to evaluate functional ability by measuring lower body strength. The chair will be placed against a wall to secure safety. The participant will sit in the middle of the seat with the feet placed flat on the floor with a shoulder width apart. The arms will be crossed at the wrists and held close to the chest. The test will begin in a sitting position. For 30 s, the participant stands completely up and then completely back down. The score is the number of completed chair stands in 30 s [40].

##### Physical activity

Information on physical activity is collected using the physical activity and vital signs questionnaire [33] and accelerometers (Activity AX3, Newcastle, UK) measuring 7 days from baseline and 7 days from 3-month follow-up.

#### Frailty

Frailty is determined using the FRAIL scale. The FRAIL scale consists of five components each containing a simple question. The components include Fatigue, Resistance, Ambulation, Illnesses, and Loss of weight. The answers will either give 0 or 1 point. A total score of 0 will categorize the participant as robust, while a total score of 1–2 and 3–5 will categorize the participant as pre-frail and frail, respectively [32].

#### Prognostic data

Information regarding oxygen therapy and exacerbations will be obtained from participants at 1- and 3-month follow-ups supplemented with information from patient files. Information regarding hospital contacts and mortality will be obtained from patient files.

### Plans to promote participant retention and complete follow-up {18b}

From previous studies among individuals with severe COPD, it has been reported that it was a challenge to recruit participants due to the burden of their clinical condition [41]. Therefore, it is important to minimize the burden for the patients and to secure a comfortable atmosphere. This study intends to have as few outcomes as possible that require physical activity from the participant, few different persons involved in patient contact, and to do all assessments as home visits. We expect this will increase the likelihood of a higher recruitment and retention rate throughout the study period. The study coordinator will be involved in every contact with the participant to secure a familiar and comfortable atmosphere. In case the study coordinator is unable to attend, it is necessary that a replacement will be arranged in agreement with the participant. The potential replacement will receive thorough training. The study coordinator is therefore involved in every step from recruitment, throughout the course of the intervention and at all visits. Participants may withdraw from the study at any time. The study coordinator will make any reasonable effort to complete the follow-up. Participants who wish to withdraw will be asked if they want to withdraw completely or partially. Partial withdrawal may be either to accept the use of their data collected prior to withdrawal or to continue without any future contact i.e. only data collection via patient files.

### Data collection for participants who discontinue or deviate from intervention protocols

Deviation from the intervention is defined as less than 6 regular contacts and an intake of less than 80% of the calculated protein or energy requirement for ≥ one third of the intervention period. Reasons for non-adherence will be registered as part of the regular contact or at follow-up visits. It is the intention to collect all data independent of deviation from the protocol.

### Data management {19}

At all visits, data will be entered directly into the electronic database REDCap (https://redcap.regionh.dk/). Though in cases with a limited internet connection or other technical issues, data will be collected on REDCap-generated case report forms and entered into REDCap as soon as possible. Access to the electronic database requires an access permit and a password.

### Confidentiality {27}

The processing of personal data will be handled in accordance with the General Data Protection Regulations. An identification number will be assigned to all included patients and all data will be handled pseudoanonymized. An identifiable list containing the project identification number will be conducted and stored in the hospital database in a network drive separately from all other project documents. The data management is estimated to end by December 31, 2025.

### Plans for collection, laboratory evaluation, and storage of biological specimens for genetic or molecular analysis in this trial/future use {33}

This project does not include the collection of biological specimens.

## Statistical methods

### Statistical methods for primary and secondary outcomes {20a}

#### Main analysis

Data on the primary outcome as well as all continuous secondary and other outcomes will be analyzed using linear mixed models that include an intervention group-time interaction as a fixed-effects term whereas between-participant variation is captured by means of (participant-specific) random (intercept) effects. There will also be random effects for the blocks used to account for the block randomization [42]. Baseline values will be part of the outcome [43].

#### Sensitivity analysis

Additionally, linear mixed models that also include relevant covariates (as fixed effects) will be fitted to adjust for observed baseline imbalance (if needed). Relevant covariates include age, BMI, sex, pulmonary function, and the number of comorbidities.

For all fitted linear mixed models, the two estimated mean changes from baseline to 3 months (for both intervention groups) as well as the estimated mean difference in change from baseline to 3 months will be reported together with the corresponding 95% confidence intervals and *p* values. Statistical significance will be declared whenever the *p*-value is below 0.05.

Where appropriate, outcome values will be transformed prior to the statistical analysis.

For categorical secondary or other outcomes data will be analyzed by means of logistic mixed-effects regression models that include an intervention group-time interaction as a fixed-effects term and block and participant-specific random effects. Baseline values will be part of the outcome. The estimated difference in change from baseline to 3 months will be reported as a ratio of odds ratios together with the corresponding 95% confidence interval and *p* value.

Statistical analyses will be carried out using STATA/IC version 17.0 (StataCorp LP, USA). Specifically, the commands XTMIXED and XTMELOGIT will be used for fitting linear mixed models (using residual maximum likelihood estimation) and logistic mixed-effects regression models (using maximum likelihood estimation), respectively.

### Interim analyses {21b}

There is no plan of conducting an interim analysis in this study.

### Methods for additional analyses (e.g., subgroup analyses) {20b}

We plan to conduct subgroup analysis based on disease burden/history and social factors including co-existing cardiovascular diseases, undernutrition, and social situation (e.g., living alone). It is expected that individuals with COPD and coexisting cardiovascular diseases or undernutrition deviate from individuals with COPD but without these conditions. Subgroup analyses will be carried out using linear or logistic mixed-effects models that include an intervention group-subgroup-time three-way interaction as a fixed-effects and randomization blocks and participants as random (intercept) effects. Subgroup effects will be reported as estimated mean differences of differences in changes from baseline to 3 months accompanied by the corresponding 95% confidence intervals and *p* values.

### Methods in analysis to handle protocol non-adherence and any statistical methods to handle missing data {20c}

#### Missing outcomes values

For the primary outcome missing outcome values will be addressed using three approaches. We will consider two approaches that use imputation, making different assumptions about participants with missing data: 1) HRQoL remains unaltered (corresponding to last observation carried forward) and 2) HRQoL deteriorates over time with a rate of 2.5% or 5% per month to a maximum deterioration of 10 and 20%, respectively. The argument for the second approach is that it is expected that the main reason for missing data will be the worsening of COPD or other comorbidities or the detection of new conditions. The first and second approaches make explicit assumptions about the missingness mechanism, which is assumed to be missing not at random. For both approaches, multiple imputations will be applied to accommodate the uncertainty related to imputing missing data and Rubin’s rule will be applied to obtain standard errors [44]. The third approach will be fitting mixed models without any imputation, using all available data (an available-case analysis), corresponding to assuming the missingness mechanism is missing at random (optimistically assuming that participants dropping out or with missing data of other reasons will have similar trajectories after drop-out/event causing missing data as the other participants). The two imputation approaches will produce estimates that may be used for evaluating effectiveness whereas the third approach will produce efficacy estimates.

For the secondary outcomes, missing outcome values will be handled in the same way as the primary outcome: (1) secondary outcomes remain unaltered after drop-out (corresponding to the last observation carried forward), and (2) weight, BMI, fat-free mass, fat mass, functional capacity (stand chair test and grip strength), and disease-related quality of life deteriorate over time with a rate of 2.5 or 5% per month to a maximum deterioration of 10 and 20%, respectively. The secondary outcomes related to hospital contacts will increase over time by 2.5 and 5% per month to a maximum of 10 and 20%, respectively. For the secondary outcomes, available-case analyses will also be carried out.

For the other outcomes, only the last observation carried forward and available-case analyses will be carried out.

Intermittent missing outcome values occurring at the 1-month visit will not be imputed.

#### Missing covariate data

Imputation of missing covariate data will be carried out using multiple imputations through chained equations (MICE) [45, 46]. However, this will only be relevant for models including covariates.

#### Non-adherence

For the augmented standard of care arm, an indicator of non-adherence will be defined if the following 2 criteria are met: < 6 regular contacts and an intake of < 80% of the calculated protein and energy requirement for at least one third of the intervention period. It will not be possible to make comparisons between the two intervention arms. However, changes within the augmented standard of care arm will be estimated using a logistic mixed-effects model including time as a fixed effect and blocks and participants as random effects. The estimated changes in non-adherence from baseline to 1 and 3 months will be reported as odds ratios together with the corresponding 95% confidence intervals and *p* values.

### Plans to give access to the full protocol, participant-level data, and statistical code {31c}

This is considered the full protocol. Thus, there are no plans for granting further access to the study protocol. Data and statistical codes may be available on request in accordance to the General Data Protection Regulations (https://intranet.regionh.dk/regi/organisation/informationssikkerhed/dataanmeldelser-og-databehandleraftaler/Sider/default.aspx).

## Oversight and monitoring

### Composition of the coordinating center and trial steering committee {5d}

Not applicable.

### Composition of the data monitoring committee, its role and reporting structure {21a}

Not applicable.

### Adverse event reporting and harms {22}

All unintended and adverse events will be registered and reported directly to the National Committee on Health Research Ethics in Denmark and to included and potential participants. Since this study is non-invasive and only includes the intake of nutritional products, we do not expect trial-related adverse events or side effects. Hospital admission and mortality are expected outcomes regardless of allocation and this outcome will be reported as part of the study.

### Frequency and plans for auditing trial conduct {23}

Not applicable.

### Plans for communicating important protocol amendments to relevant parties (e.g. trial participants, ethical committees) {25}

If important changes or modifications to the protocol are considered it will be notified and approved by National Committee on Health Research Ethics in Denmark.

### Dissemination plans {31a}

All results (positive, negative, and inconclusive) will be disclosed and submitted for publication in international peer-reviewed journals and presented as posters or oral presentations at relevant national and international conferences. The results will be reported in accordance with the Consolidated Standards of Reporting Trials (CONSORT) guidelines [47]. Results relevant for the management of the patients will be made available for clinicians and we will seek influence on guidelines. Authorship will be based on the Vancouver recommendations.

## Discussion

Despite growing evidence suggesting that nutritional therapy should have more attention, nutrition is still a neglected area in the outpatient care of COPD patients likely due to lacking knowledge and the absence of professional responsibility [14]. It has been estimated that 25–40% of individuals with COPD are undernourished [48]. Consequences related to undernutrition in COPD include frequent and prolonged hospital admissions, declining functional capacity and ability to carry out daily activities, higher risk of pressure ulcers and infections, fatigue, and apathy with a negative influence on appetite, reduced ability to cough resulting in an increased risk of lower respiratory tract infection such as pneumonia, heart failure, social isolation and depression [49]. Weight stability and prevention of weight loss are possible, but it requires professional responsibility and a patient-centered approach. We have designed a patient-centered, nutritional intervention. We will investigate the effects in a randomized controlled trial. We expect that the experience from the study and the results will provide insight into the importance of patient-centered nutritional care strategies as part of outpatient care for individuals with severe COPD.

Living with severe COPD is associated with a high burden of symptoms, especially in the late stages of the disease and it has been reported that symptom relief is often limited. Many individuals with severe COPD experience decreasing HRQoL especially in physical, psychiatric, and social domains [50]. The combination of limited symptom relief and mobility may result in a vicious cycle of frailty, fatigue, and social isolation [51]. At the end stage of COPD, many individuals are afraid or unable to leave their homes, and many are highly dependent of assistance [51]. This further increase social isolation which may result in anxiety, panic and depression again negatively influencing HRQoL. At the end stage of the disease, mental health and HRQoL are the most essential outcomes from the individual perspective. At this stage, it is a fact that death has become closer. It is essential to focus on the quality of life and mental health at this stage to provide a more acceptable life at the end-stage of COPD.

The standard of care at the outpatient clinic at Copenhagen University Hospital—North Zealand focuses on basic palliative care for individuals with severe COPD. The structure of the outpatient care program is designed to be able to respond to the individual and changing needs of the patients and it has resulted in improved HRQoL [52]. However, despite the patient-centered approach with an increased focus on individual palliative needs, these patients still have impaired HRQoL. The current study built on this strategy to further improve HRQoL at the end-stage of COPD.

If the study demonstrates the expected improvements in HRQoL, nutritional status, and prognosis, this may be a step closer to ensuring an improved and worthier end-of-life for individuals with severe COPD.

## Trial status

The National Committee on Health Research Ethics in Denmark approved the 2nd version of the study protocol on February 15, 2021. The trial was registered at www.ClinicalTrials.gov on May 3, 2021. The first patient-first visit was on May 25, 2021, and inclusion is expected to be completed in March 2023.

## Data Availability

The project group will have access to the final data set. There are no contractual agreements that limit access.

## Abbreviations

COPD: Chronic obstructive pulmonary disease
HRQoL: Health-related quality of life
BMI: Body mass index
NRS2002: Nutritional Risk Screening 2002
MNA: Mini Nutritional Assessment
